# Humoral and Cellular Immune Responses Induced by Bivalent DNA Vaccines Expressing Fusion Capsid Proteins of Porcine Circovirus Genotypes 2a and 2b

**DOI:** 10.3390/vaccines12030324

**Published:** 2024-03-18

**Authors:** Sochanwattey Meas, Khuanjit Chaimongkolnukul, Jaraspim Narkpuk, Phenjun Mekvichitsaeng, Kanokwan Poomputsa, Nanchaya Wanasen, Yaowaluck Maprang Roshorm

**Affiliations:** 1 School of Bioresources and Technology, King Mongkut’s University of Technology Thonburi, Bangkok 10150, Thailand; sochanwattey.m@mail.kmutt.ac.th (S.M.); kanokwan.poo@kmutt.ac.th (K.P.); 2 National Laboratory Animal Center, Mahidol University, Nakhon Pathom 73170, Thailand; khuanjit.cha@mahidol.edu; 3 National Center for Genetic Engineering and Biotechnology (BIOTEC), National Science and Technology Development Agency (NSTDA), Pathum Thani 12120, Thailand; jaraspim.nar@biotec.or.th (J.N.); nanchaya.wan@biotec.or.th (N.W.); 4 Pilot Plant Development and Training Institute, King Mongkut’s University of Technology Thonburi, Bangkok 10150, Thailand; phenjun.mek@kmutt.ac.th

**Keywords:** PCV2, bivalent vaccine, DNA vaccine, B cell epitope, T cell epitope

## Abstract

Porcine circovirus type 2 (PCV2) is the main causative agent of porcine circovirus-associated disease (PCVAD) that profoundly impacts the swine industry worldwide. While most of the commercial PCV vaccines are developed based on PCV genotype 2a (PCV2a), PCV genotype 2b (PCV2b) has become predominant since 2003. In this study, we developed and evaluated DNA-based bivalent vaccines covering both PCV2a and PCV2b. We generated a new immunogen, PCV2b-2a, by combining consensus sequences of the PCV2a and PCV2b capsid proteins (Cap2a and Cap2b) in a form of fusion protein. We also examined whether modifications of the PCV2b-2a fusion protein with a signal sequence (SS) and granulocyte macrophage-colony stimulating factor (GM-CSF) fusing with interleukine-4 (IL-4) (GI) could further improve the vaccine immunogenicity. An immunogenicity study of BALB/cAJcl mice revealed that the DNA vector pVAX1 co-expressing PCV2b-2a and GI (pVAX1.PCV2b-2a-GI) was most potent at inducing both antibody and cellular immune responses against Cap2a and Cap2b. Interestingly, the vaccines skewed the immune response towards Th1 phenotype (IgG2a > IgG1). By performing ELISA and ELISpot with predicted epitope peptides, the three most immunogenic B cell epitopes and five putative T cell epitopes were identified on Cap2a and Cap2b. Importantly, our DNA vaccines elicited broad immune responses recognizing both genotype-specific and PCV2-conserved epitopes. Sera from mice immunized with the DNAs expressing PCV2b-2a and PCV2b-2a-GI significantly inhibited PCV2a cell entry at serum dilution 1:8. All these results suggest a great potential of our PCV2b-2a-based vaccines, which can be further developed for use in other vaccine platforms to achieve both vaccine efficacy and economical production cost.

## 1. Introduction

Porcine circovirus (PCV) is a small, nonenveloped virus with a diameter ranging from 17 to 22 nm, containing a single-stranded (ss), circular DNA genome and belonging to the family Circoviridae [1]. PCV2 is the major causative agent of several syndromes and diseases collectively named as porcine circovirus-associated disease (PCVAD), which includes post-weaning multisystemic wasting syndrome (PMWS) or systemic disease, porcine dermatitis, and nephropathy syndrome (PDNS), as well as porcine respiratory disease complex (PRDC), congenital tremors type II (CT), and reproductive failure [2]. PCVAD is an emerging disease in swine and currently is becoming one of the most economically crucial diseases threatening the swine industry worldwide.

PCV2 viruses are divided into six genotypes, PCV2a, PCV2b, PCV2c, PCV2d, PCV2e and PCV2f [3]. However, two additional genotypes, PCV2g and PCV2h, which are potentially recombinant forms, have also been proposed [4]. The PCV2 genome has two main open reading frames: ORF1 and ORF2 [5]. ORF1 encodes the viral replicase proteins, Rep and Rep’, while ORF2 encodes the viral capsid protein which contains antigenic epitopes of the virus [5]. From the early 1990s until 2000, PCV2a was the most widely circulating genotype in circulation; however, PCV2b has become more predominant from 2003 onwards [6]. PCV2 genotypes share an amino acid similarity of about 97–100% for Rep and 89–100% for the capsid protein [7]. Retrospective studies have reported that PCV2a was predominant on pig farms with and without PMWS, while PCV2b has been found commonly on farms with PMWS outbreaks [8]. Some studies have highlighted that PCV2b and PCV2a are pathogenically comparable [9] and appear to be of equivalent virulence [10], while a field study suggests that PCV2b is more virulent [11]. Interestingly, many studies have reported the detection of co-existence infections with various PCV2 genotypes in the same pig. The study on 118 PCV2-positive DNA samples of pigs with PCVAD using a modified differential PCR assay showed that the co-existence rate of PCV2 was 32.2% (38/118), in which 12 out of 38 were further confirmed by DNA sequencing to have co-existence between PCV2a and PCV2b [12]. Importantly, dual heterologous inoculation of PCV2a and PCV2b resulted in a more acute clinical illness in pigs [10].

To control PCV2 infection, several commercially available PCV2 vaccines have been developed mainly based on PCV2a. Although the PCV2a-based vaccines have been demonstrated to provide cross-protection to PCV2b [13], it has been reported that immunization with a bivalent PCV2a-2b vaccine provides better results for prevention against distinct PCV2 genotypes compared to a monovalent vaccine. Pigs that received a bivalent chimeric PCV2a-PCV2b live vaccine exhibited reduced percentages of viremic individuals, less lymphoid depletion and/or histiocytic replacement, and individuals with PCV2 colonization of lymphoid tissues, compared to monovalent either PCV2a or PCV2b vaccine [14]. Given the desire for a PCV2a-PCV2b bivalent vaccine, most of the bivalent vaccines tested are a mixture of two monovalent vaccines, which require separate preparation and validation of the two vaccines, leading to high cost and time consumed. Thus, alternative methods such as combining the protein antigens from two different genotypes into one fusion form may address these issues while maintaining vaccine efficacy.

For the development of PCV vaccines, ORF2-encoded capsid protein, the only one structural protein of PCV, is the main target for the development of PCV recombinant vaccines as it contains immunogenic epitopes of the virus [15,16]. For an effective protection against PCV, both humoral and cell-mediated immune responses in a sufficient amount are required [17]. One of the methods to achieve a high magnitude of immune response is co-immunization with cytokines. Granulocyte macrophage-colony stimulating factor (GM-CSF) and interleukine-4 (IL-4) are among the top cytokines that have been extensively studied for co-administration with vaccines. GM-CSF is a glycosylated cytokine with the size of 26 kDa and is secreted by various cell types including T and B lymphocytes, macrophages, endothelial cells, and fibroblasts [18,19]. GM-CSF has been reported as a stimulator to activate antigen-presenting cells (APCs) including dendritic cells (DCs), which play a crucial role in the induction of T cell immune responses as well as antibody responses [20]. On the other hand, interleukin-4 (IL-4) was described as a B cell stimulator factor that mediates B cell activation and proliferation [21]. GM-CSF and IL-4 have been deployed either individually or synergistically as molecular adjuvants in immunization to augment the immune responses [22,23,24].

As CD4 T cell and B cell generally recognize and are stimulated by exogenous antigens, a vaccine capable of providing both endogenous and exogenous antigen in host cell system should induce not only CD8 T cells but also enhance CD4 T and B cell stimulation. For the vaccine platforms that generally generate an endogenous antigen such as DNA and adenoviral vectors, targeting an immunogen to secretory pathway for extracellular secretion is one of the methods that can promote the generation of exogenous antigens by the transfected/transduced cells. Enhancement of CD4 (helper) T cell response may in turn enhance B and CD8 T cell responses as CD4 T (T helper) cells are involved in the stimulation of both B and CD8 T cells [25]. Previous studies showed that the inclusion of a secretory signal sequence in the immunogen enhanced the induction of immune responses when compared to the non-secretory forms [26,27]. Thus, it is useful to address whether a PCV2 capsid-based immunogen would be more immunogenic in a secreted form.

In this work, we developed and evaluated a new bivalent PCV2 vaccine against PCV2a and PCV2b. We designed a new bivalent immunogen combining capsid protein of PCV2a and PCV2b as a fusion protein. The bivalent immunogen was further examined with regards to whether an addition of signal sequence (SS) to the N-terminus and the fusion protein of GM-CSF and IL-4 (GI) to the C-terminus could improve the vaccine immunogenicity. With its ease of preparation and capacity to induce both humoral and cellular immune response, DNA vector pVAX1 was our choice for the development of bivalent PCV2 vaccines. Immunogenicity of the recombinant pVAX1 vectors carrying the immunogen gene *PCV2b-2a* and its derivatives were tested in BALB/cAJcl mice. Both humoral and cellular immune responses were then measured. Antibody responses were measured using ELISA, while neutralizing activity was determined using a neutralization assay. T cell responses were investigated using ELISpot.

## 2. Materials and Methods

### 2.1. Protein Sequence Retrieval and Phylogenetic Analysis

Amino acid sequences of the PCV2a and PCV2b capsid proteins were retrieved from the National Center for Biotechnology Information (NCBI). Sublime Text program (Version 2.0) was employed to eliminate irrelevant and incomplete amino acid sequences. SeaView program (vVersion 9.20) was used for multi-alignment by using Muscle, followed by grouping the strains using the phylogenetic tree, based on distance analysis, NJ method, ignore gap, and bootstrap 1000 replication. After grouping, PCV2a and PCV2b groups were assigned according to the typical motif sequences [28]. Consensus sequences were generated from the PCV2a and PCV2b groups using Unipro UGENE v1.25.0 (32-bit version) (Supplementary Figure S1). A new bivalent PCV2 immunogen was then created by combining the consensus Cap2a and Cap2b into a fusion protein, designated PCV2b-2a (Supplementary Figure S2).

### 2.2. Prediction of the Immunogen’s Properties

The physicochemical properties (such as molecular weight, isoelectric point (PI), half-life, and stability) of the consensus PCV2a and PCV capsid proteins and the designed immunogen were predicted using the ExPASy ProtParam server “https://web.expasy.org/protparam/ (accessed on 6 March 2024)”. The antigenicity property of the immunogen proteins was in silico evaluated using the Vexigen v2.0 “https://www.ddg-pharmfac.net/vaxijen/VaxiJen/VaxiJen.html (accessed on 6 March 2024)”. The tertiary structure of the immunogen was predicted using the ColabFold v1.5.5: AlphaFold2 online server “https://colab.research.google.com/github/sokrypton/ColabFold/blob/main/AlphaFold2.ipynb (accessed on 6 March 2024)”. The PyMOL program v.2.5.2 was used for visualization of the 3D model and epitope labelling.

### 2.3. B Cell Epitope Prediction

Immunoinformatics tools were exploited to predict linear B cell epitopes and properties of the amino acid residues. The prediction in this study followed the tools and prediction approach described by Polyiam et al. [29,30]. The prediction is based on a combination of the result from multiple tools provided in iedb database “http://tools.iedb.org/bcell/ (accessed on 3 March 2020)” including BepiPred (prediction of B cell epitopes and coil structure), Emini (surface accessibility prediction), Kolaskar and Tongaonkar (antigenicity prediction), and Parker (hydrophilicity prediction). IUPred2 “https://iupred2a.elte.hu/ (accessed on 3 March 2020)” was used to predict flexibility. Only the peptides with the length of at least 6 continuous residues predicted by each tool were selected and subjected to further analysis. Prediction was performed with the consensus sequences of the PCV2a and PCV2b capsid proteins. Selected epitopes were subjected to peptide synthesis (Mimotopes, Mulgrave, Australia).

### 2.4. CD8 T Cell Epitope Prediction

Two immunoinformatics tools, NetMHC and NetCTLpan, were employed to predict CD8 T cell epitopes. NetMHC-4.0 “http://www.cbs.dtu.dk/services/NetMHC/ (accessed on 3 March 2020)” predicts the binding of peptides to MHC class I molecules. Putative epitopes were selected based on percentile ranking score lower than 0.5 (<0.5) and binding affinity below 500 nM (<500 nM) to swine MHC class I alleles (SLA) and mouse MHC class I alleles (H-2). On the other hand, NetCTLpan-1.1, which predicts cytotoxic T lymphocyte (CTL) epitopes based on peptide MHC class I binding, proteasomal C terminal cleavage, and TAP transport efficiency “http://www.cbs.dtu.dk/services/NetCTLpan/ (accessed on 3 March 2020)” were also employed to predict CD8 T cell epitope from the PCV2a and PCV2b capsid proteins. Putative epitopes were selected based on MHC class I binding score > 0.54 and combined score > 0.74 to swine SLA class I alleles and mouse H-2 alleles. CD8 T cell epitopes were chemically synthesized (Mimotopes).

### 2.5. Generation of DNA Vaccine

The amino acid sequences of the bivalent immunogen PCV2b-2a and the fusion protein of GM-CSF and IL-4 (GI) were converted to nucleotide sequences, codon-optimized with pig codon usage and synthetically made (IDT, Singapore). The fusion gene *GI* was fused to the bivalent gene *PCV2b-2a* using overlapping PCR, by which the sequence encoding self-cleave peptide FuF2A was added between the two constructs via primers (5′-flanking-RE-F vs. V5(PCV)FuF2A-R and V5(PCV)FuF2A-F vs. Stop-RE-R) giving rise to a new gene construct, designated *PCV2b-2a-GI*. Furthermore, the signal peptide (SS) sequence was included into the two bivalent genes via primers (SS-PCV2b-F1, SS-PCV2b-F2, PstI-SS-F3 vs. Stop-RE-R) during PCR amplification, thus giving rise to 2 more gene constructs, namely *SS-PCV2b-2a* and *SS-PCV2b-2a-GI*. Primers used in the PCR amplification are shown in Table 1. PCR amplification was performed using 2× Phusion Green HS II HF Master Mix (Thermo Scientific, Waltham, MA, USA) according to the manufacturer’s instruction. Digested and PCR products of the bivalent gene fragments were gel purified using Universal DNA purification kit (TIANGEN, Beijing, China) and ligated into a mammalian expression plasmid pVAX1 (Invitrogen, Waltham, MA, USA) at the PstI and NotI sites. *E. coli* DH5α was transformed with the ligated plasmids using the standard heat shock method. Recombinant clones were grown on LB agar containing kanamycin (50 ng/μL) and screened by rapid size screening. Plasmids were extracted from selected clones and subsequently verified by restriction enzyme digestion and automated DNA sequencing. Plasmid in a large-scale preparation was then extracted from a chosen clone of each construct using EndoFree Plasmid Mega Kits (Qiagen, Hilden, Germany) and used for protein expression study and immunization.

### 2.6. Preparation of the PCV2 Capsid Proteins

To prepare antigen for immune response study, the synthetic genes encoding PCV2a and PCV2b capsid protein (in a separate gene fragment) were digested with restriction enzymes HindIII and NotI (both from NEB, Frankfurt am Main, Germany), gel purified and inserted into an *E. coli* expression plasmid pET28b (Invitrogen) digested with the same restriction enzymes. Recombinant clones were first selected in *E. coli* DH5α on LB agar containing kanamycin (50 ng/μL). After the sequence was confirmed by automated DNA sequencing, the plasmid was next used for transforming *E. coli* BL21(DE3)pLysS. The chosen clones were grown in LB medium containing kanamycin. When OD600 reached 0.4–0.6, gene expression was induced with 1 mM IPTG (Isopropyl β-d-1-thiogalactopyranoside (Himedia, Maharashtra, India)). After a 3-h incubation at 37 °C, the cells were collected, resuspended with PBS, and disrupted using a sonicator. After centrifugation at 10,000 rpm for 15 min, the supernatant was transferred to a new tube and stored at −20 °C. The presence of capsid proteins in the cell lysate was confirmed by Western blot using anti-V5 antibody as a primary antibody (Supplementary Figure S3). The proteins obtained from this step was used as antigen in Indirect Enzyme-Linked Immunosorbent Assay (ELISA).

### 2.7. Cell Culture and Transfection

Human embryonic kidney (HEK) 293A cells (Invitrogen, Catalog number: R70507) were cultured in Dulbecco modified Eagle medium (DMEM) (Sigma, St. Louis, MO, USA) supplemented with 10% fetal bovine serum (FBS, HyClone, Logan, UT, USA) and 1% pen/strep antibiotics (Sigma) and incubated at 37 °C with 5% CO_2_. The cells grown in a 12-well plate were transfected with 1 µg of the plasmid pVAX1 carrying the bivalent immunogen genes using polyethylenimine (PEI, Sigma) with the plasmid:PEI ratio of 1:1. Following a 48-h incubation at 37 °C with 5% CO_2_, medium and cells were collected for protein detection using immunofluorescence staining and Western blot.

### 2.8. Immunofluorescence Staining

After a 2-day incubation, transfected HEK-293A cells in a 12-well plate were fixed with 3.7% formaldehyde for 10 min and then washed once with PBS, prior to permeabilizing with 90% cold methanol for 5 min at 4 °C. The cells were washed once with PBS and blocked with PBS containing 2% FBS (*v*/*v*) (2% FBS/PBS) overnight at 4 °C. The 2% FBS/PBS solution was replaced by primary antibodies, mouse anti-V5 (Invitrogen) diluted 1:2000 in PBS, or goat anti-IL-4 (R&D systems, Minneapolis, Minnesota, MN, USA) diluted 1:500 in PBS and incubated for 2 h at room temperature. The cells were washed twice with PBS, followed by an addition of the secondary antibodies, donkey anti-mouse IgG conjugated with Alexa Fluor^®^ 594 (Abcam, Cambridge, UK) diluted 1:1000 in PBS, or anti-goat IgG conjugated with NL493 (R&D systems) diluted 1:500 in PBS. After an incubation for 1 h at room temperature. The cells were washed twice with PBS and stained for the nuclei with 4′,6-diamidino-2-phenylindole (DAPI, Sigma) at a final concentration of 2 µM in PBS at room temperature for 5–10 min. Following a wash with PBS, the cells were photographed under an inverted fluorescence microscope (Olympus IX73, Tokyo, Japan) with the 20× objective lens.

### 2.9. Western Blot Analysis

Forty-eight hours after transfection, culture medium was collected and the transfected HEK-293A cells were lysed with 200 µL of cold lysis buffer RIPA (20 mM Tris, [pH 8.0], 137 mM NaCl, 10% (*v*/*v*) glycerol, 1% (*v*/*v*) Nonidet P-40). Protein samples were mixed with 4× sample loading buffer (150 mM Tris-HCl, pH 6.8, 35% (*v*/*v*) glycerol, 10% (*w*/*v*) SDS, 4% (*v*/*v*) Triton X-100, 400 mM DTT, 0.4% (*w*/*v*) bromophenol blue) with a ratio 1:4 and then heated at 95 °C for 5 min. Proteins were then separated on 10% SDS-PAGE and transferred by electroblotting onto a nitrocellulose membrane (Bio-Rad, Hercules, CA, USA) using Trans-Blot^®^ SD Semi Dry Electrophoretic Transfer Cell (Bio-Rad). The membranes were blocked with PBS containing 5% (*w*/*v*) skim milk and 0.1% (*v*/*v*) Tween 20 (5% PBST) overnight at 4 °C and subsequently incubated with a 1:2000 diluted mouse anti-V5 antibody (Invitrogen) or a 1:2000 diluted goat anti-IL-4 antibody (R&D systems) for 2 h at room temperature. Membranes were washed 3 times with PBS containing 0.1% (*v*/*v*) Tween 20 (PBST), followed by an incubation with a 1:10,000 diluted horseradish peroxidase (HRP)-conjugated goat anti-mouse IgG antibody (Abcam) or a 1:4000 diluted HRP-conjugated donkey anti-goat IgG antibody (R&D systems). In parallel, an internal control staining was also performed by using a 1:2000 diluted mouse anti-human PCNA (eBioscience, San Diego, CA, USA) as a primary antibody and a 1:10,000 diluted HRP-conjugated goat anti-mouse IgG antibody (Abcam) as a secondary antibody. Note that all primary and secondary antibodies are diluted in 5% PBST. Following a 2-h incubation with secondary antibody at room temperature, the membranes were washed 3 times with PBST, incubated with SuperSignal^TM^ West Pico PLUS chemiluminescent substrate (Thermo Scientific) for 5 min, and photographed using the C-DiGit Blot Scanner (LI-COR Bioscience, Lincoln, NE, USA).

### 2.10. Mouse Immunization

Animal experiments were carried out under the regulation and permission of the National Laboratory Animal Center (NLAC), Mahidol University, IACUC protocol number RA2022-16. Female 6-week-old BALB/cAJcl mice were purchased from Nomura Siam International Co., Ltd, Bangkok, Thailand. Five mice of each group were intramuscularly (i.m.) injected into thigh muscles of both hind limbs with 100 µg of recombinant DNA diluted in 100 µL of PBS. Recombinant DNA used for injection were pVAX1.PCV2b-2a, pVAX1.SS-PCV2b-2a, pVAX1.PCV2b-2a-GI, and pVAX1.SS-PCV2b-2a-GI. In addition, a group of five female BALB/cAJcl mice was injected with PBS (100 µL) and used as a negative control group. Mice were immunized twice with a 3-week interval. Blood and spleen samples were collected 3 weeks after the second immunization.

### 2.11. Indirect Enzyme-Linked Immunosorbent Assay (ELISA)

A 96-well microplate (Greiner bio-one) was coated with antigens, either 0.5 nmole of synthetic epitope peptides (for epitope profiling study) or 5 µg of crude protein extract of the PCV2a and PCV2b capsid proteins produced by *E. coli* (for determining titers of IgG, IgG1 and IgG2a). For determination of total IgG titers, individual Cap2a and Cap2b were used, while determination of the IgG1 and IgG2a titers used a mixture of Cap2a and Cap2b (2.5 µg each). Note that the amount of crude protein extract used in ELISA assay (5 µg/well) had been previously tested to determine an optimal amount for the assay (Supplementary Figure S4). The plates were incubated overnight at 4 °C with the antigen diluted in 50 µL PBS per well. The plates were washed twice with PBS containing 0.05% Tween 20 (PBST) (200 µL/well) and then blocked with 5% FBS (HyClone) diluted in PBST (150 µL/well). After a 1-h incubation at room temperature, the serum samples were diluted in PBST containing 1% FBS to a 1:100 dilution or 2-fold serial dilution, added into each well, and incubated for 1 h at room temperature. Following a 3-time wash with PBST (200 µL/well), secondary antibodies diluted 1:10,000 in PBST containing 1% FBS were added to the wells. These include goat anti-mouse IgG-HRP, goat anti-mouse IgG1-HRP, and goat anti-mouse IgG2a-HRP (all from Abcam). The plates were incubated at room temperature for 30 min. After 3 washes with PBST (200 µL/well), 70 µL of TMB substrate solution (BioLegend, San Diego, CA, USA) was added into each well and incubated in the dark for 15 min at room temperature and the reaction was finally stopped by adding 30 µL of 2 N sulfuric acid (H_2_SO_4_) into each well. Optical density was then read at wavelength 450 nm using a microplate reader (Thermo scientific). Endpoint titer was determined based on the mean OD450 + 2SD calculated from all dilutions of the PBS group. The last serum dilution that gave OD450 value greater than this cutoff was recognized as endpoint titer of that sample.

### 2.12. IFN-γ Enzyme-Linked Immunosorbent Spot (IFN-γ ELISpot)

Spleen samples collected in 10 mL of complete RPMI (RPMI 1640 medium (Himedia) supplemented with 10% (*v*/*v*) FBS (HyClone) and 1% pen/strep antibiotics (Sigma)) were mashed with a syringe plunger on a cell strainer (70 µm) in a petri dish. After a centrifugation at 1500× *g*, 4 °C for 3 min, the cell pellet was resuspended with 5 mL of cold red blood cell lysis buffer (eBioscience) to lyse red blood cells. Following a centrifugation and a wash with cold PBS, the cell pellet was resuspended with 3 mL of complete RPMI. The splenocytes were then subject to IFN-γ ELISpot assay. ELISpot was performed using murine IFN-γ ELISpot kit (ImmunoSpot, Cleveland, OH, USA), following the manufacturer’s instruction. Briefly, ELISpot plates were pre-activated with 70% ethanol, followed by coating with murine IFN-γ capture antibody and incubating at 4 °C overnight. After washing once with PBS, splenocytes (2 × 10^5^ cells/well in 100 µL) mixed with peptides (1 nmole/well in 100 µL of complete RPMI) were added into each well. The well without peptide was used as a negative control. After an incubation at 37 °C, 5% CO_2_ for 36 h, plates were washed twice with PBS, followed by a 2-time wash with PBS containing 0.05% Tween 20 (PBST). Diluted biotinylated anti-murine IFN-γ detection antibody was added into each well, followed by an incubation at room temperature for 2 h. After washing with PBST three times, diluted streptavidin-AP was added into each well. Following an incubation for 30 min, the plates were washed twice with PBST and further washed twice with distilled water. The plates were then incubated with a developing solution to allow distinct spots to emerge and then stopped by gently rinsing with distilled water. Spot forming cell (SFC) was read and counted using CTL ImmunoSpot^®^ S6 Universal Analyzer (ImmunoSpot) and ImmunoSpot software (ImmunoSpot 7.0.9.4 Professional Analyzer DC).

### 2.13. Serum Neutralization Assay

Functional ability of antibodies to prevent infection of virus in vitro was tested using neutralization assay based on fluorescent foci reduction as previously described [31]. Serum samples were heat inactivated at 56 °C for 30 min prior to performing the serum neutralization assay. All mouse serum samples were 2-fold serially diluted in 50 µL per well with Minimum Essential Medium (MEM, HyClone) to the dilution ranging from 1:8 to 1:1024. PCV2a virus was diluted in 50 µL per well with MEM to obtain 100 TCID_50_. Subsequently, 50 µL of diluted serum was mixed with 50 µL of diluted PCV2a and incubated at 37 °C. Negative control was set by incubating the virus with MEM. One hour-post incubation, 100 µL of the mixture was transferred into 96-well plates containing approximately 70% confluent monolayer of PK-15 cells and incubated at 37 °C with 5% CO_2_. Each condition was performed in duplicate. After a 3-h incubation, the medium was removed, replaced with new MEM supplemented with 5% (*v*/*v*) FBS (Sigma) and pen/strep antibiotics (Gibco, Grand Island, NY, USA), and further incubated at 37 °C with 5% CO_2_. At 48 h post-infection, immunofluorescence staining was performed to determine PCV2a-infected cells. Briefly, the cells were washed once with PBS, fixed with ice cold 80% (*v*/*v*) acetone and incubated at room temperature for 15 min. Following a wash with PBS, the cells were blocked with 2% (*w*/*v*) BSA in PBS for 30 min at room temperature. Blocking buffer was removed and replaced with a 1:1000 diluted rabbit anti-PCV2a antibody in dilution buffer (PBS with 0.05% Tween 20 and 1% BSA). After a 1-h incubation, cells were washed 4 times with PBST (PBS with 0.05% Tween 20). A 1:1000 diluted goat anti-rabbit IgG conjugated with Alexa fluor 488 (Abcam) in dilution buffer was added into the cells and incubated for 1 h at room temperature. After a 4-time wash with PBST, cells were mounted with antifade mounting medium containing DAPI (Vector Laboratories, Newark, CA, USA). Stained cells were captured by Opera Phenix High-Content Screening System (PerkinElmer, Waltham, MA, USA) and the fluorescent spots were directly examined and counted by Columbus Server using the cell count function.

### 2.14. Statical Analysis

Statical analysis was performed using GraphPad Prism version 8.0.1 for Windows (GraphPad Software, San Diego, CA, USA). The statical significance of ELISA results against predicted linear B cell epitopes was analyzed by one-way ANOVA of SPSS 23 for window (SPSS Software, Chicago, IL, USA). A value of *p* < 0.05 was considered statically significant. Immunodominant epitopes were identified based on Z-score of differential OD450 (Dif. OD), which are calculated with the formula shown below.

Dif. OD (of each peptide in individual mouse) = OD450 of certain peptide in each immunized mouse − OD450 mean of certain peptide in the PBS group

Dif. OD mean = average of the Dif. OD of all peptides in all immunized mice

Z-score = Dif. OD of each peptide in individual mouse − Dif. OD mean

A peptide showing mean of Z-score greater than 0.1 (>0.1) was defined as immunodominant epitope.

## 3. Results

### 3.1. Sequence Retrieval, Analysis and Generation of the PCV2a and PCV2b Capsid Consensus Sequences

Amino acid sequences of the PCV2a and PCV2b capsid proteins (Cap2a and Cap2b) were retrieved from NCBI in FASTA formats (June 2017). These amino acid sequences were further verified in Sublime Text3 program. After a removal of irrelevant or incomplete sequences, 450 accession numbers were obtained for Cap2a and Cap2b. The sequences were grouped based on sequence similarity using phylogenetic tree tool in SeaView program. Groups of the PCV2a and PCV2b genotypes were identified based on the typical motif 86-TNKISI-91 present in Cap2a and the motifs 86-SNPRSV-91 present in Cap2b [28], respectively. The analysis revealed 43 and 260 amino acid sequences for Cap2a and Cap2b, respectively. We then aligned these amino acid sequences of each genotype using MUSCLE alignment tool in Unipro UGENE program and generated consensus sequences (Supplementary Figure S1), which were further used in immunogen design and epitope predictions.

### 3.2. Design and In Silico Validation of Bivalent PCV2 Immunogens

**Figure 1:**
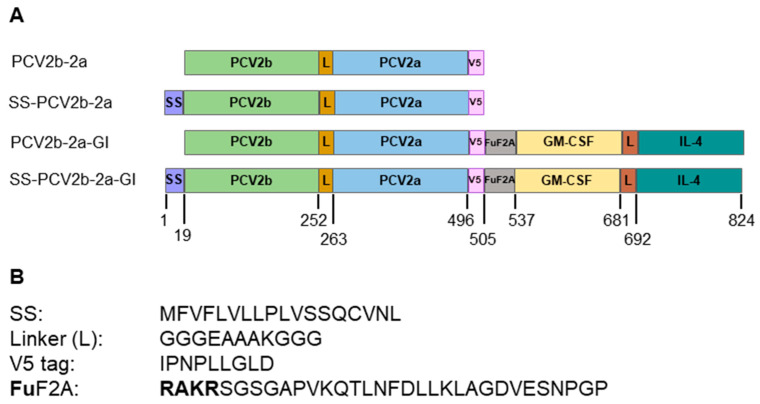
Schematic representation of four newly designed immunogens and additional sequences added to the immunogens. (**A**) Schematic representation of four newly designed immunogens. A new immunogen, PCV2b-2a, was designed by linking the capsid from PCV2b and PCV2a together. An alpha helical linker (L) was added between the two proteins and V5 tag (V5) was added to the C-terminus of the immunogen. The immunogen was further modified with signal sequence (SS) and mucosal adjuvants, GM-CSF fused with IL-4 (GI), giving rise to 4 PCV2b-2a constructs: PCV2b-2a, SS-PCV2b-2a, PCV2b-2a-GI, and SS-PCV2b-2a-GI. Furin cleavage site linked with self-cleaving peptide F2A (FuF2A) was added to the upstream of the GI. The numbers represent amino acid residues in the constructs. (**B**) Extra sequences added to the immunogen. The furin cleavage site (Fu; RAKR) is indicated with bold letters.

A new bivalent PCV2 immunogen was designed by joining consensus amino acid sequences of Cap2a to the C-terminus of Cap2b, designated PCV2b-2a. The two proteins are linked together via an alpha helical linker to allow a separation of the two proteins to maintain their functions [32,33]. V5 tag sequence was fused to the C-terminus of the fusion protein as shown in Figure 1 and Supplementary Figure S2. With the aim to maximize potency of the PCV2b-2a immunogen, we further modified the immunogen with the signal sequence (SS) and molecular adjuvants GM-CSF and IL-4. To target the protein to secretory pathway, a signal sequence derived from SARS-CoV-2 spike (accession no. MN996527.1), which was previously used to mediate extracellular secretion of our immunogens with high success, was fused to the N-terminus of the immunogens. Porcine GM-CSF and IL-4 were combined as a fusion protein (GI) with an alpha helical linker connecting the two proteins. The fusion protein GI was added to the C-terminus of the PCV2b-2a immunogen. A self-cleaving peptide derived from foot-and-mouth disease virus protein, F2A [34], with a furin (Fu) cleaving sequence (RAKR) [34,35] that we previously demonstrated as having high efficiency in mediating self-cleavage of co-expressed PRRSV proteins [36], was included between the immunogen and GI. The F2A mediates self-cleavage of the co-expressed immunogen and GI, while Fu site mediates trimming of the F2A from the immunogen. With all these modifications, we generated 4 bivalent immunogen constructs: (i) PCV2b-2a, (ii) SS-PCV2b-2a, (iii) PCV2b-2a-GI, and (iv) SS-PCV2b-2a-GI (Figure 1).

Prediction of the physico-chemical property demonstrates that the designed immunogen Cap2b-2a has 486 amino acid residues with molecular weight of 57.5 kDa and an isoelectric point (PI) of 10.68. The predicted half-life is 30 h in mammalian reticulocytes, more than 20 h in yeast, and more than 10 h in *E. coli*. Its instability index value is 57.35, which is greater than 40, indicating that the protein is unstable. The predicted aliphatic index of 64.12 indicates that the protein is thermostable. The predicted GRAVY (grand average of hydropathicity) (hydrophobicity index) of −0.816 suggests that the protein is hydrophilic. In addition, the result of the Vaxigen v2.0 analysis demonstrates that the immunogen Cap2b-2a is a protective antigen with the antigenicity score of 0.6041, which is greater than the threshold of 0.4. These properties of the PCV2b-2a immunogen resemble those of the natural PCV2a and PCV2b capsid proteins (Supplementary Table S1). The tertiary structure prediction reveals that the 3-D structures of the consensus Cap2a and Cap2b proteins resemble the structures of the natural PCV2 capsid protein reported in the database (PDB 3R0R) (Supplementary Figure S5, suggesting that the consensus amino acids do not disrupt conformation of the PCV2 capsid protein. When the consensus Cap2a and Cap2b linked together as a fusion protein, the structures of these two proteins are well preserved.

### 3.3. Generation of Bivalent DNA Plasmids and Protein Expression Study

**Figure 2:**
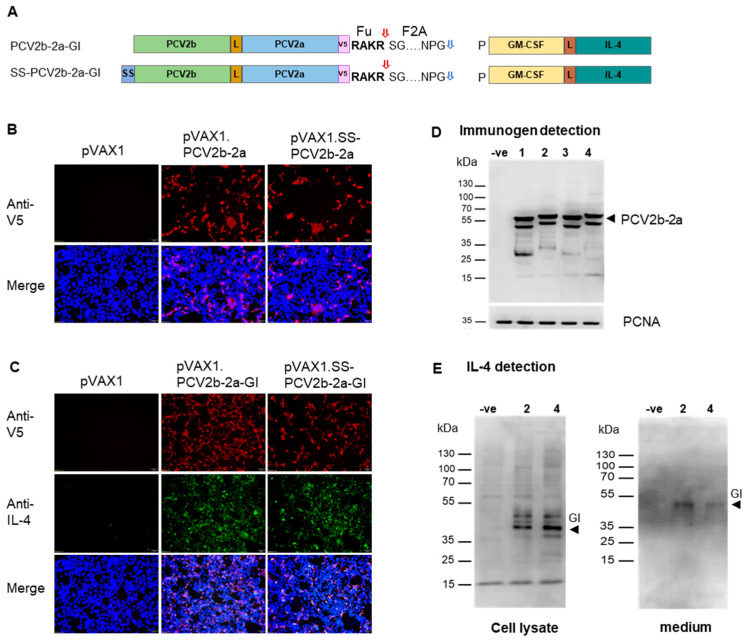
Expression of the immunogen by 4 different constructs. HEK-293A cells were transfected with 1 µg of the pVAX1 plasmids harboring 4 different immunogen constructs. Cells and medium were collected and tested for protein expression at 48 h post-transfection. (**A**) Schematic diagram of the protein products produced by the constructs bearing the fusion gene *GI*. Proteins PCV2b-2a and GI are cleaved during translation, which is mediated by F2A. Red arrow indicate furin cleavage site; blue arrow indicates F2A cleavage site. (**B**) Immunofluorescence assay detecting the PCV2b-2a immunogen expressed by the two constructs: PCV2b-2a and SS-PCV2b-2a. (**C**) Immunofluorescence assay detecting the immunogen and fusion cytokine GI expressed by the two constructs: PCV2b-2a-GI and SS-PCV2b-2a-GI. (**D**) Western blot analysis of the PCV2b-2a fusion protein expressed by all 4 constructs in the cell lysate. PCNA was also detected and used as an internal control. (**E**) Western blot analyses of the fusion cytokine GI expressed from the two constructs bearing the fusion gene *GI*. Both cell lysate and medium were tested. The PCV2b-2a, GI and PCNA proteins were detected using anti-V5 mAb, anti-IL-4 mAb, and anti-PCNA mAb, respectively, in both immunofluorescence staining and Western blot. Cell nuclei were stained with DAPI (blue fluorescence). In Western blot, the cells transfected with the plasmid pVAX1 expressing PCV2b-2a, PCV2b-2a-GI, SS-PCV2b-2a, and SS-PCV2b-2a-GI are indicated as 1, 2, 3, and 4, respectively. The HEK-293A cells transfected with parental plasmid pVAX1 were used as a negative control (-ve).

Amino acid sequences of the immunogens were converted to nucleotide sequences and codon-optimized with pig codon usage to maximize protein production. Kozak sequence (GCCACCATG) was added to the upstream of coding region of the immunogen genes to enhance gene expression. The genes, *PCV2b-2a* and *GI*, were chemically synthesized. To generate *PCV2b-2a*-derivative genes, the *SS* and *GI* fragments were added to the *PCV2b-2a* gene construct via PCR amplifications. All 4 gene constructs were then inserted into a mammalian expression plasmid pVAX1. We next performed in vitro expression of the recombinant DNAs by transient transfection in the HEK-293A cell line. As the two immunogens, PCV2b-2a-GI and SS-PCV2b-2a-GI, contain furin cleavage site and self-cleaving peptide (FuF2A), which mediate cleavage of the upstream and downstream proteins, they will give a product of 2 discrete proteins: PCV2b-2a and GI (Figure 2A). The 2 fusion proteins, PCV2b-2a and GI, were detected using anti-V5 and anti-IL-4, respectively. Immunofluorescence staining with anti-V5 antibody revealed that cells transfected with the DNAs of all four constructs, but not empty pVAX1, showed clear red fluorescent signal dispersing all over the well (Figure 2B,C). Co-staining with anti-V5 and anti-IL-4 antibodies exhibited the presence of PCV2b-2a (red fluorescence) and GI (green fluorescence) in the cells transfected with the DNAs bearing *PCV2b-2a-GI* and *SS-PCV2b-2a-GI* gene (Figure 2C), indicating a successful co-expression of PCV2b-2a and GI.

The proteins were further investigated using Western blot. The PCV2b-2a protein expressed by pVAX1.PCV2b-2a and pVAX1.SS-PCV2b-2a was detected at the size of approximately 57.5 kDa as expected, while pVAX1.PCV2b-2a-GI and pVAX1.SS-PCV2b-2a-GI gave PCV2b-2a with a larger size of about 61 kDa due to the presence of the FuF2A peptide at the C-terminus (Figure 2D). For the constructs bearing signal sequence (SS-PCV2b-2a and SS-PCV2b-2a-GI), the cell culture medium was also tested using Western blot to investigate extracellularly secreted PCV2b-2a. However, we could not detect the protein in the medium of both constructs. The GI fusion cytokine expressed by the constructs PCV2b-2a-GI and SS-PCV2b-2a-GI was detected using anti-IL-4 antibody but the size was slightly higher than the expected size of 32 kDa in both whole cell lysate and culture medium (Figure 2E), which is possibly due to post-translational modifications. The presence of the 61-kDa Cap2b-2a band (Figure 2D) and GI protein in the medium (Figure 2E) indicates a successful cleavage of these two proteins mediated by FuF2A. Taken together, the results indicate that the PCV2b-2a protein could be expressed by all four constructs and the PCV2b-2a and GI proteins were successfully co-expressed and cleaved to discrete forms under the presence of the FuF2A peptide.

### 3.4. B Cell and CD8 T Cell Epitope Prediction

**Figure 3:**
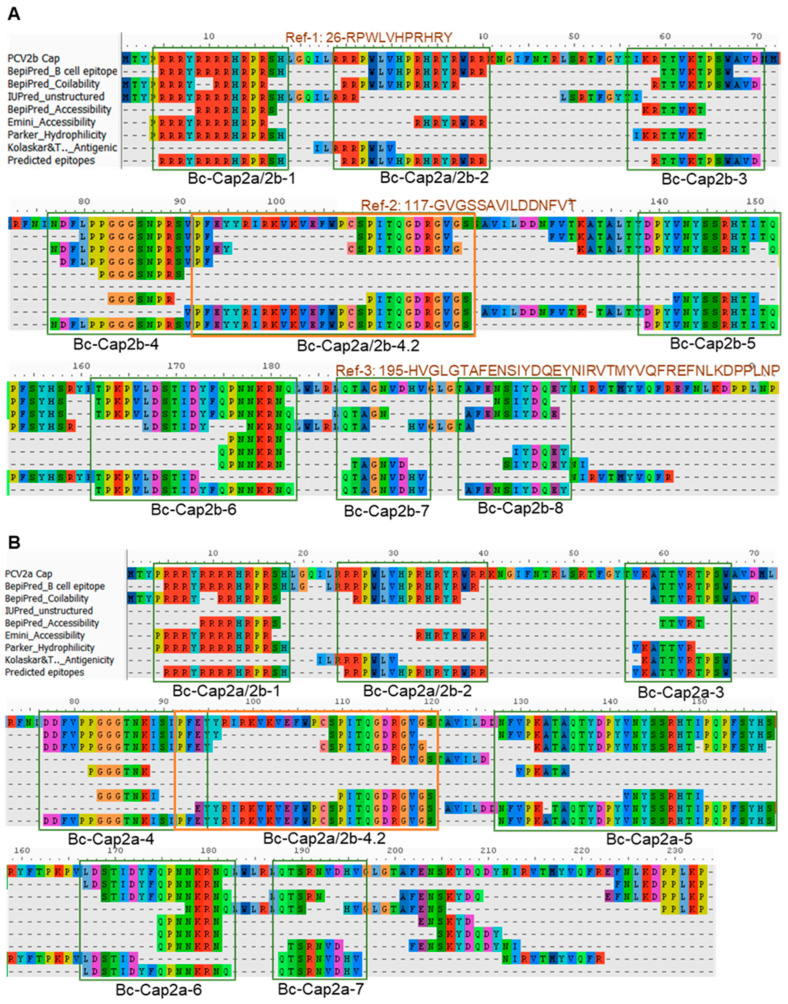
B cell epitope prediction of the Cap2a and Cap2b proteins. The consensus sequences of Cap2a and Cap2b were subjected to B cell epitope prediction using immunoinformatics approach. (**A**) Prediction result of Cap2b. (**B**) Prediction result of Cap2a.

B cell epitopes were predicted from the PCV2a and PCV2b capsid consensus sequences using the immunoinformatics method described by Polyiam et al. [29,30]. From the prediction, 10 peptides in 8 regions of Cap2b (Figure 3A, Table 2) and 8 peptides in 7 regions of Cap2a (Figure 3B, Table 2) were in silico characterized as linear B cell epitopes. Peptides were synthesized according to the sequences of predicted epitopes. Three published B cell epitopes of the PCV2 capsid were used as reference epitopes. These include Ref-1 (26-RPWLVHPRHRY-36) [37], Ref-2 (117-GVGSSAVILDDNFVT-131) [16], and Ref-3 (195-HVGLGTAFENSIYDQEYNIRVTMYVQFREFNLKDPPLNP-233) [38]. Their locations on the PCV2 capsid protein are shown in Figure 3A.

To enable T cell response study, CD8 T cell epitopes were predicted and chemically synthesized. The prediction was performed with the consensus sequence of the Cap2a and Cap2b proteins and based on both swine (swine leukocyte antigen: SLA) and mouse (H-2) MHC class I alleles. By using NetCTLpan-1.1 and NetMHC-4.0 for the prediction, a total of 17 SLA-I-restricted peptides and 3 H-2^d^-restricted peptides were predicted with high potential to be CD8 T cell epitopes and they were subjected to peptide synthesis and used in immunoassay (Table 3).

### 3.5. Mouse Immunization and Total IgG Response

**Figure 4:**
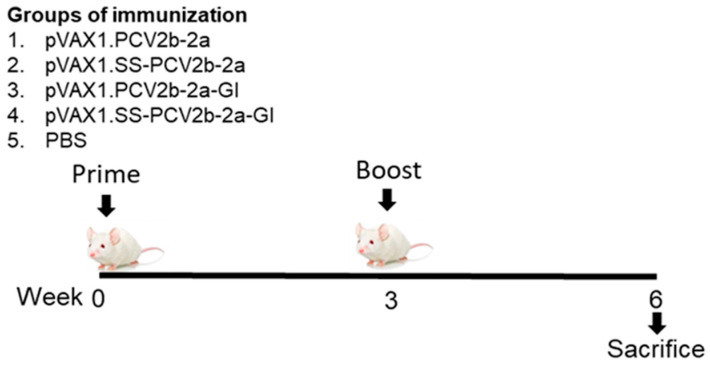
Immunization protocol. Groups of 6-week-old female BALB/cAJcl mice (5 mice/group) were injected intramuscularly with 100 μg of the pVAX1 DNA bearing four different immunogen genes as indicated.

Five groups of female 6-week-old BALB/cAJcl mice (5/group) were immunized via intramuscular injection with 100 µg of the DNA vaccines or PBS as a control (Figure 4). All mice received one booster injection with the same DNA on day 21. On day 42, mice were sacrificed and blood and spleen samples were collected. Sera were prepared from blood and analyzed by ELISA to determine antibody response (humoral immune response).

**Figure 5:**
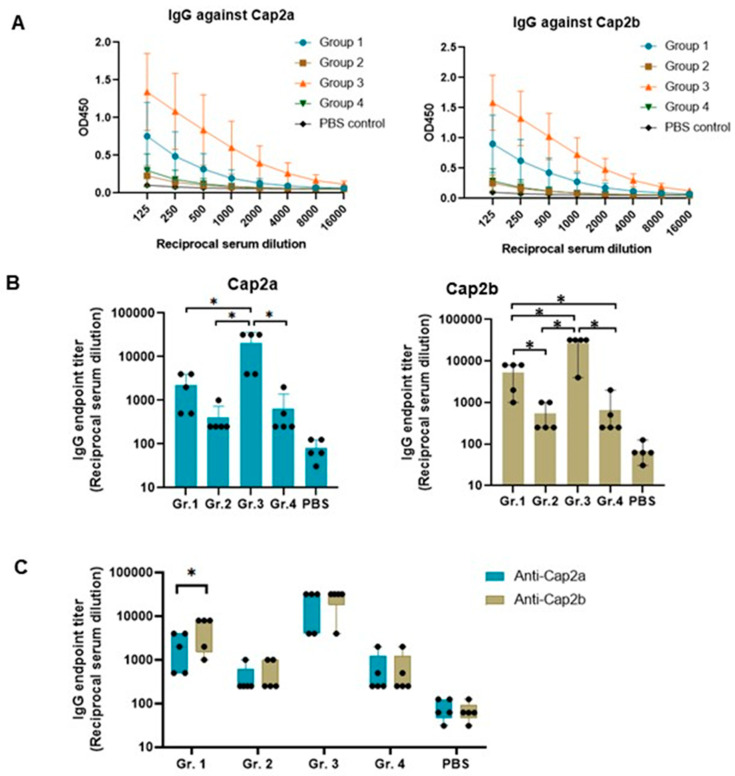
Total IgG responses against capsid proteins of PCV2 genotype 2a and 2b. ELISA was performed with a 2-fold serially diluted serum. The recombinant proteins Cap2a and Cap2b in *E. coli* lysate were used as antigens. (**A**) Reactivity of total IgG in the serum samples at different dilutions with Cap2a and Cap2b as antigens. OD450 means of each dilution are shown with standard deviations. (**B**) Endpoint titers of total IgG against Cap2a (left panel) and Cap2b (right panel). (**C**) Comparison of the IgG endpoint titers against Cap2a and Cap2b in each immunization group. Black dot (●) in (**B**,**C**) represents endpoint titer of individual mouse. Asterisk (*) indicates significant difference (*p* < 0.05).

Endpoint titer of total IgG antibody was examined using capsid proteins, Cap2a and Cap2b, expressed individually in *E. coli* (Supplementary Figure S3). The optimal amount of protein used for coating plates was studied and 5 µg protein/well was chosen to be used in ELISA (Supplementary Figure S4). A 2-fold serial dilution of serum samples (1:125–1:16,000) was tested in ELISA (Figure 5A). Mice immunized with pVAX1.PCV2b-2a-GI (Gr. 3) exhibited the highest Cap2a- and Cap2b-specific antibody responses with endpoint titer means of 20,800 and 26,400, respectively, followed by the group immunized with pVAX1.PCV2b-2a (Gr. 1) with IgG endpoint titer means against Cap2a and Cap2b of 2200 and 5400, respectively (Figure 5B). The immunogen constructs, SS-PCV2b-2a (Gr. 2) and SS-PCV2b-2a-GI (Gr. 4), induced a much lower total IgG response with endpoint titer means against Cap2a and Cap2b both lower than 700. Based on OD450 mean, three groups of immunized mice (groups 1 to 3) exhibited higher antibody response against Cap2b than Cap2a, although only the response in group 1 was statistically significant (*p* < 0.05) (Figure 5C).

### 3.6. Th1/Th2 Immune Response Induced by the PCV2b-2a-Based DNA Vaccines

**Figure 6:**
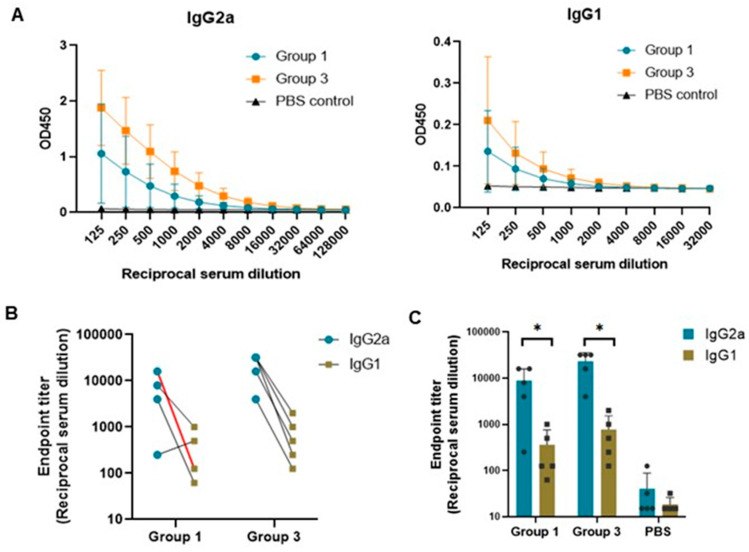
IgG subtype response. Antibody subtypes IgG2a and IgG1 were investigated using ELISA. Sera from mice immunized with pVAX1.PCV2b-2a (group 1) and pVAX1.PCV2b-2a-GI (group 3) were used in the assay in parallel with the PBS control group. ELISA was performed with 2-fold serially diluted sera, by which a mixture of the proteins Cap2a and Cap2b was used as an antigen. (**A**) OD450 mean with standard deviation of the IgG2a and IgG1 in 2-fold serially diluted sera. (**B**) Comparison of the IgG2a and IgG1 endpoint titers in each mouse of groups 1 and 3. In group 1, the red line indicates endpoint titers of 2 mice sharing the same value. (**C**) IgG2a and IgG1 endpoint titers in groups 1 and 3 in comparison to the PBS control group. Means of endpoint titer (color bar) with standard deviations of each group as well as endpoint titer of each mouse (Black dot (●)) are shown. Asterisk (*) indicates significant difference (*p* < 0.05).

Further, we assessed whether the immune response induced by the two immunogen constructs, PCV2b-2a (Gr. 1) and PCV2b-2a-GI (Gr. 3), is Th1- or Th2-biased, which is indicated by IgG2a and IgG1, respectively [39]. We performed ELISA using anti-mouse IgG2a and IgG1 as secondary antibodies with 2-fold serially diluted sera. The result showed that the OD450 of IgG2a were markedly higher than that of IgG1 with a IgG2a:IgG1 ratio as high as 128 in several mice of both groups (Figure 6A). Endpoint titers of the IgG2a were in the range of 250 to 32,000, while those of the IgG1 was in the range of 62 to 2000 (Figure 6B,C). These results strongly confirm that PCV2b-2a-based DNA vaccines, regardless of the presence of the GI fusion cytokine, skewed immune responses towards a Th1 phenotype.

### 3.7. Linear B Cell Epitope Profile of Cap2a and Cap2b

**Figure 7:**
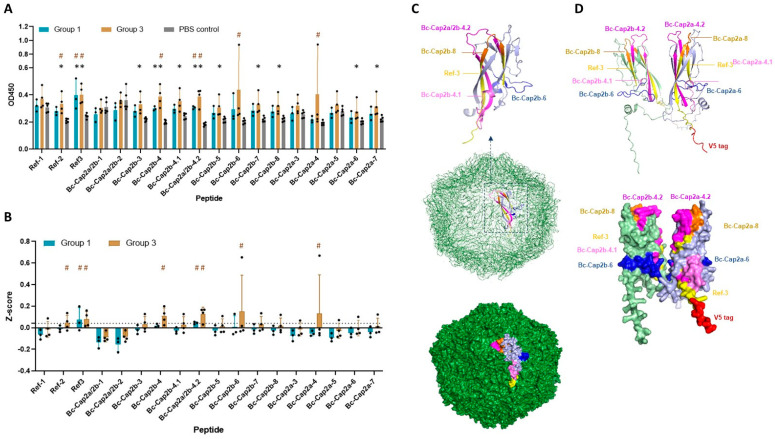
Antibody responses against predicted linear B cell epitopes and determination of immunodominant epitopes. (**A**) IgG response against individual predicted linear B cell epitopes. (**B**) Determination of immunodominant epitopes based on Z-score. Black dot (●) in (**A**,**B**) represents value of individual mouse. Serum samples (diluted 1:100) from mice immunized with pVAX1.PCV2b-2a (group 1), pVAX1.PCV2b-2a-GI (group 3), and the PBS control group were tested with synthetic peptides corresponding to the predicted linear B cell epitopes of Cap2a and Cap2b as indicated in *X*-axis. Antibody responses from each mouse are shown. Ref-1 to Ref-3 are published epitopes and used as referenced epitopes. A peptide showing Z-score mean > 0.04 (cutoff indicated by dashed line (--)) was considered immunodominant. Asterisk (*) indicates a significant difference between immunized group and PBS control group (*p* < 0.05). Octothorpe (#) indicates immunodominant epitope. (**C**) Three-dimensional structure of the natural PCV2 capsid protein (PDB 3R0R) showing in monomeric form (**top** panel) and assembled PCV2 capsid (**middle** panel) with surface exposure (**bottom** panel). (**D**) Three-dimensional structure of the immunogen PCV2b-2a showing secondary structure component (**top** panel) and surface exposure (**bottom** panel). The immunodominant epitopes are depicted on both proteins with different colors.

The synthetic peptides corresponding to the predicted linear B cell epitopes were tested for their reactivity with mouse sera. In this assay, sera from mice group 1 (pVAX1.PCV2b-2a) and group 3 (pVAX1.PCV2b-2a-GI) were tested in comparison to sera from the PBS control. Reactivity of the peptides with their target antibodies was determined based on statical analysis comparing OD450 value of vaccine-immunized mice to that of the PBS control mice (*p* < 0.05). As shown in Figure 7A, statistical analysis revealed that 9 predicted peptides showed reactivity with at least one group of the immunized mice. The response against the reference peptides, Ref-2 and Ref-3, in immunized mice was also significantly higher than that of the control group. Regardless of the statistical difference, immunodominant epitopes were determined based on the Z-score described in Materials and Methods. Predicted epitopes Bc-Cap2b-4, Bc-Cap2a/2b-4.2, Bc-Cap2b-6, and Bc-Cap2a-4 as well as 2 reference epitopes Ref-2 and Ref-3 were categorized as the most immunoreactive or immunodominant epitopes (Figure 7B). However, all these immunodominant epitopes are clustered in 3 main regions of the PCV2 capsid protein including the following: (i) epitope region 4, residues 77–120 (Bc-Cap2b-4, Bc-Cap2a/2b-4.2, Bc-Cap2a-4, and Ref-2); (ii) epitope region 6, residues 162–182 (Bc-Cap2b-6); and (iii) epitope region 8, residues 195–233 (Ref-3). Thus, we conclude that B cell epitopes located at these 3 regions of the PCV2 capsid protein were most immunogenic in the context of our PCV2b-2a immunogen. Remarkably, the antibody response against each peptide was considerably low with OD450 mostly ranging from 0.2 to 0.5, while detection with the recombinant capsid proteins gave OD450 as high as 2 in several serum samples, suggesting a contribution of capsid-specific antibodies induced by conformational epitopes on Cap2a and Cap2b.

Locations and secondary structures of the most immunogenic linear B cell epitopes on the natural monomeric PCV2 capsid protein and assembled PCV2 capsid (virion) (PDB 3R0R) (Figure 7C and Supplementary Figure S5) and the PCV2b-2a immunogen (Figure 7D) are shown. The secondary structure composition of the immunodominant epitopes on the natural PCV2 capsid protein are similar to that on the PCV2b-2a immunogen. All these immunodominant epitopes contain parts that are exposed on the capsid surface (Figure 7C,D), suggesting accessibility by B cell receptors and antibodies, which are critical for B cell stimulation and virus neutralization.

### 3.8. Cellular Immune Response and CD8 T Cell Epitope Profile

**Figure 8:**
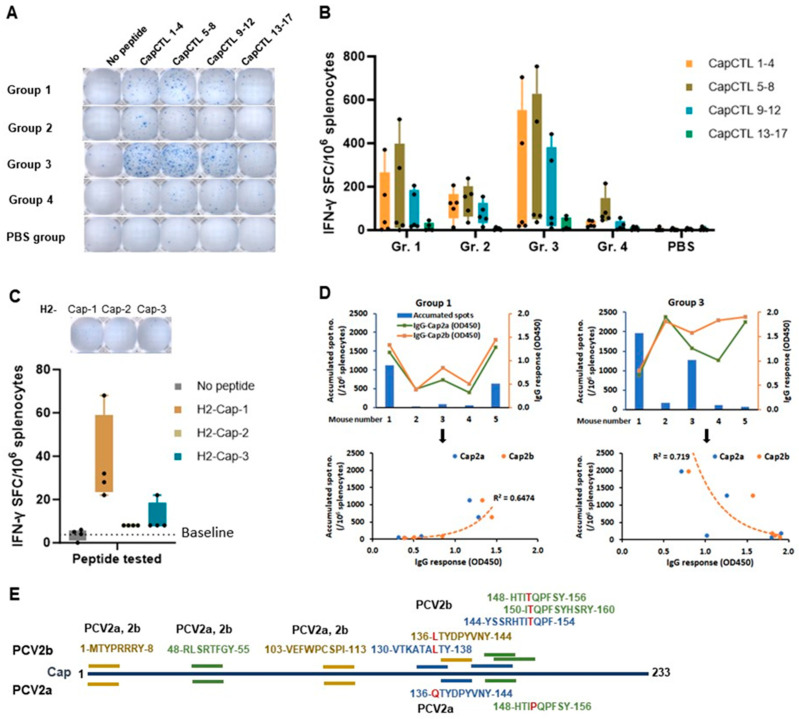
Cellular immune responses in immunized mice. IFN-γ ELISpot was performed by re-stimulating the splenocytes with either single or pooled peptides of predicted CD8 T cell epitopes. (**A**) Representative of ELISpot wells of each group obtained from re-stimulating the splenocytes with four CapCTL peptide pools. (**B**) Frequencies of IFN-γ-producing cells (spot-forming cell (SFC) in response to CapCTL peptide pools). (**C**) Frequencies of IFN-γ-producing cells in response to single peptides, H2-Cap-1 to 3. Spot number in each group of figures (**B**,**C**) is presented in box and whisker plot, by which the middle 50% of scores for the group is indicated in box. Black dot (●) represents spot number of individual mouse. (**D**) Correlation of the T cell response and antibody response (total IgG) of groups 1 and 3. Accumulated spot number (in primary *Y*-axis) and total IgG (presented as OD450) against Cap2a and Cap2b (in secondary *Y*-axis) of each mouse in the groups (*X*-axis) are shown. (**E**) Position and sequence of putative CD8 T cell epitopes. Peptides with potential to be CD8 T cell epitopes were obtained by subtracting peptides in reactive pools (pools CapCTL-1–4 (orange), 5–8 (green), and 9–12 (blue)) with non-reactive pool (CapCTL-13–17) and H2-Cap peptides that share amino acid residues and are located at the same positions. Letter in red indicates a residue that is different between Cap2a and Cap2b.

IFN-γ ELISpot assay was employed to examine frequency of responding CD8 T cells. Splenocytes were incubated for 36 h with 4 SLA-restricted peptide pools (CapCTL1–4, CapCTL 5–8, CapCTL 9–12, and CapCTL 13–17) and 3 single H-2-restricted peptides (H2-Cap-1, H2-Cap-2, and H2-Cap-3) obtained from prediction. When re-stimulated with SLA-restricted peptide pools, splenocytes from all 4 immunized groups showed spots with various numbers (Figure 8A). In agreement with the humoral immune response results, the highest T cell immune response was observed in mice immunized with pVAX1.PCV2b-2a-GI (group 3), followed by group 1 (pVAX1.PCV2b-2a) (Figure 8A,B). Consistent in all immunized groups, the frequency of IFN-γ-expressing cells was highest when the cells were re-stimulated with the CapCTL 5–8 peptide pool, followed by CapCTL 1–4, and CapCTL 9–12, respectively, (Figure 8A,B). Peptide pool CapCTL 13–17 was least immunogenic as only a few cells produced IFN-γ in response to this pool. When tested with individual H-2 predicted peptides, only a small proportion of the cells produced IFN-γ when incubated with peptide H2-Cap-1 (Figure 8C). The results of the T cell immune response and antibody response (total IgG) of groups 1 and 3 were combined in one graph for a comparison and determine the correlation as shown in Figure 8D. Interestingly, these two types of immune responses are directly correlated in group 1, but inversely correlated in group 3 (Figure 8D and Supplementary Figure S6). By subtracting overlapping peptides in the CapCTL 13–17 peptide pool and three single H-2 peptides from those in the CapCTL 5–8, CapCTL 1–4, and CapCTL 9–12 pools (immunogenic pools), we identified 5 regions on the Cap2a and Cap2b that are potentially CD8 T cell epitopes. These include peptides in the following residues: (i) 1–8, (ii) 48–55, (iii) 103–111, (iv) 130–144, and (v) 144–160 (Figure 8E). However, further verification with a single peptide is required to define the exact sites and sequences of genuine immunodominant CD8 T cell epitopes.

### 3.9. Neutralizing Activity of the Antibodies from Immunized Mice

**Figure 9:**
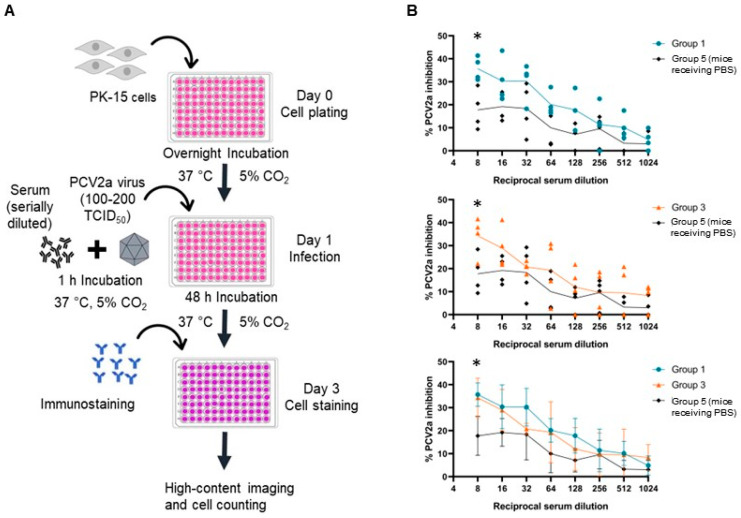
Antibody neutralization assay against PCV2a. (**A**) Schematic representation of antibody neutralization assay against PCV2a. Serum samples collected from different groups of immunized mice were 2-fold serially diluted in MEM to the dilution ranging from 1:8 to 1:1024 and incubated with a predetermined amount of PCV2a (100 TCID_50_) for 1 h at 37 °C. Subsequently, the virus–serum mixtures were added to PK-15 cells. The cells were incubated for 48 h, after which the infection was analyzed by immunofluorescence staining. The fluorescence spots were directly captured via Opera Phenix High-Content Screening System and counted via Columbus Server using the cell count function. The assay was conducted in technical duplicates. (**B**) Inhibition percentage of serially diluted sera against PCV2a viruses. Inhibition by each serum sample of groups 1 and 3 are shown in the two top panels with mean value at each dilution. Means with standard deviations of each group are shown (bottom panel). Asterisk (*) indicates a significant difference between the immunized group and the PBS control group (*p* < 0.05).

Neutralizing activity of the antibodies from immunized mice was examined using a neutralization assay by testing with infectious PCV2a. PK-15 cells were infected with a mixture of infectious PCV2a virus and a 2-fold serially diluted serum. Non-infected PK-15 cells were used as a negative control. PCV2a infection was determined by immunofluorescence staining. The details of the neutralization assay are summarized in Figure 9A. Endpoint titers of the neutralizing antibodies were determined based on the statistical analysis (*p* < 0.05) at the dilution that % PCV2a inhibition of the immunized groups is higher than 20% and significantly different from that of the PBS control group. As shown in Figure 9B, sera from mice receiving pVAX1.PCV2b-2a (Group 1) and pVAX1.PCV2b-2a-GI (Group 3) both presented neutralizing antibody titers of 8.

## 4. Discussion

In the present study, we designed a new bivalent PCV2 immunogen, PCV2b-2a, that combines the capsid protein from PCV2a and PCV2b. We found that the PCV2b-2a fusion protein profoundly induced both PCV2a- and PCV2b-specific immune responses when used as a DNA vaccine in a mouse model. Moreover, an addition of GM-CSF/IL-4 (GI) fusion cytokine further increased the vaccine immunogenicity, demonstrating that the fusion cytokine served as an effective adjuvant for improving vaccine potency.

In the protein expression study, we could not detect the immunogen containing signal sequence (SS-PCV2b-2a) secreted into the medium of transfected cell culture. A failure in targeting the protein to secretory pathway is presumably due to the presence of a nuclear signal in the PCV capsid protein. The PCV capsid protein is generally located in cytoplasm and contains a nuclear signal located in its first 42 amino acids at the N-terminus, thus allowing nuclear transport [40]. This nuclear signal may hamper the capsid protein from entering the secretory pathway as it has been demonstrated that nuclear signal-truncated PCV2 capsid protein was successfully secreted and could be mainly detected in the culture medium when it is conjugated with secretory signal peptide and induced robust immune responses in immunized mice [41].

In terms of immunogenicity, the immunogen construct PCV2b-2a-GI induced significantly higher antibody and T cell responses in mice compared to other three construct. It is known that GM-CSF regulates immune responses by activating maturation of dendritic cells [42], leading to an efficient induction of CD4 and CD8 T cells [43]. IL-4 has been shown to be involved in the development of humoral immunity, the differentiation and maturation of B cells, and the switch from IgE to IgG [25,44]. Thus, enhanced immune induction by PCV2b-2a-GI may be supported by these attributes of GM-CSF and IL-4. In agreement with our results, an immunization with the recombinant PCV2 capsid protein fused with GM-CSF increased PCV2-specific antibody production and enhanced the transcription levels of cytokines IL-2, IFN-γ, IL-4, and IL-10 in a mouse model [45]. Likewise, an immunization with a HSV-1 recombinant virus expressing IL-4 induced a humoral immune response of greater magnitude than immunization with the parental control virus [24].

In addition to measurement of IgG response, we studied Th1/Th2 response by measuring antibody markers, IgG2a and IgG1, which serve as a measure of Th1 and Th2 responses, respectively [39]. Capsid-specific antibody responses in both groups of mice receiving pVAX1.PCV2b-2a and pVAX1.PCV2b-2a-GI were primarily of the IgG2a and to a lesser extent of IgG1 isotype, suggesting a highly Th1-polarized immune response. This result indicates that immune responses with Th1 phenotypes in groups 1 and 3 are independent from the presence of GM-CSF and IL-4 in the construct. Similar to our findings, several studies on immunization with DNA vaccines demonstrated Th1-biased immune responses. A clinical study of the DNA vaccine against human papillomavirus (HPV) showed a bias towards a Th1 phenotype by stimulating T cells to produce IFN-γ, an indication of Th1 response [46]. Additionally, immunization with the DNA vaccine was more efficient at inducing the production of Th1 cytokine (IFN-γ) in immunized mice than the protein subunit vaccine as shown by a higher ratio of Th1 and Th2 cytokine (IFN-γ: IL-5) [47]. On the other hand, immunization with PCV2 capsid protein in the form of virus-like particles (VLPs) induced higher IgG1 levels than the IgG2a levels, suggesting a Th2-biased immune response [48]. From this evidence, it is likely that polarization towards Th1 and Th2 responses is greatly influenced by vaccine platforms, by which DNA vaccines that produce endogenous antigens tend to induce a greater Th1 response, while VLP/protein subunit vaccines that represent exogenous antigens tend to provide Th2-biased immune response [48,49,50]. Th1 responses have been suggested to be the preferential immune responses for the control of PCV2 infection. It was shown that the Th1 response is essential for PCV capsid-specific cytotoxic T lymphocyte production [49]. Virus-neutralizing antibody responses also displayed a direct correlation with PCV capsid-specific IgG2a levels, conveying vital roles of Th1 responses in protecting against PCV2 infection [49].

Immunodominant linear B cell epitopes of Cap2a and Cap2b in the context of our newly designed immunogen PCV2b-2a were found to be clustered in 3 main regions of Cap2a and Cap2b. Most of the B cell epitopes we identified here, except epitope Cap2a/2b-1, have been previously reported, which include epitopes in the following regions: (i) Cap2-2, Cap2-3, Cap2-4, Cap2-5, Cap2-6, Cap2-7 and Cap2-8 [15,50]; (ii) Cap2-4.1 and Cap2-4.2 [51]; (iii) Cap2-2 [37]; and (iv) Cap2-8 [38]. Based on these results, epitopes in the regions Cap2-2, Cap2-4, and Cap2-8, were most frequently shown to be immunodominant epitopes, which corresponds to our finding. Although most of the linear B cell epitopes (except Cap2a/2b-1) we identified are not novel and largely share amino acid sequences with reported epitopes, here, we demonstrated an application and efficiency of the immunoinformatics method we previously developed as described by Polyiam et al. [29,30]. In other studies, linear B cell epitopes were identified using various methods such as screening the overlapping synthetic peptides [15,50] or random peptide-displayed library [51] with monoclonal/polyclonal antibodies, which are cost- and time-consuming. Our results proved that the immunoinformatic method works as efficient as the conventional techniques with less resources and time required.

Remarkably, the antibody responses against linear B cell epitopes of the Cap2a and Cap2b proteins are considered low compared with the response detected by using the whole protein of Cap2a and Cap2b, which is consistent with the results reported by Shang et al. (2009) [50]. These results imply that a greater proportion of the antibodies elicited by the PCV2 capsid protein recognizes conformational epitopes. However, most of the reported epitopes are linear epitopes as identification of conformational epitopes is much more difficult. B cell epitope profile can be applicable in various purposes such as development of epitope-based vaccines and antibody detection kits. However, for the development of PCV vaccine, whole capsid protein may provide a superior immune induction to short peptides and chimeric multiepitope immunogen when used as antigens. As Cap2b and Cap2a in the PCV2b-2a immunogen well maintain their tertiary structures and have similar surface exposure profile to the capsid protein present on the PCV2 capsid (virus), this suggests that our immunogen can provide conformation epitopes for B cell receptor and antibody recognition.

Regarding the functions of amino acid residues within the epitope regions on virus infection and tropism, the epitope in the Cap2-4 region, which has been identified as immunodominant epitopes in a number of studies, contains the putative heparan sulfate binding site (residues 98-IRKVKV-103) [52]. Therefore, it is interesting to determine whether antibodies against this epitope can abrogate the attachment of PCV2 to the host cells. The study by Shang et al. (2009) revealed that the monoclonal antibody (3F6) recognizing epitopes in the regions Cap2-5 and Cap2-6 were capable of neutralizing PCV2 virus [50]. On the other hand, two neutralizing monoclonal antibodies, 7F5 and 6H9, were reported as having the ability to interact with conformational epitopes [50]. Similarly, monoclonal antibodies, namely 1D2, reacted only with the recombinant capsid protein, suggesting the requirement of conformational epitope [37]. The results from these studies suggest a contribution of both linear and conformational epitopes of the capsid protein on eliciting neutralizing antibodies against the PCV2 virus.

The PCV2a neutralization study showed only low neutralizing antibody titer with an endpoint titer of 8 in both groups 1 and 3 of immunized mice. This level of neutralizing antibody titer is considered low and may not be sufficient to provide a complete protection against PCV2 in pigs. This is not surprising as immunization with naked plasmid DNA is well recognized for its low immunogenicity [53]. This DNA vaccine is designed for pig usage; however, the administration of DNA vaccines in large animals requires high amounts, leading to high costs for pig producers. Thus, it may not be possible to apply a DNA vaccine for a real use in pigs and further development using other vaccine platforms such as viral vector and subunit protein is needed for both a lower cost of vaccine preparation and greater immunogenicity. Once the bivalent PCV2 vaccines have been successfully developed, they can be tested for their immunogenicity and efficacy in comparison to a commercial vaccine.

For the T cell response, three out of four peptide pools were demonstrated to be immunogenic. Although we do not know exactly which predicted epitopes are genuine CD8 T cell epitopes, subtraction analysis revealed five regions on Cap2a and Cap2b that potentially harbor immunodominant CD8 T cell epitopes. Four T cell epitopes in the immunogenic pools in our study overlap with the immunogenic T cell epitope of the PCV2 capsid protein previously identified by Stevenson et al. (2007) using 20-mer overlapping peptides and the lymphocyte proliferation method [54]. Among these immunogenic peptides, peptides at residues 131–150 and 141–160, which completely cover our epitopes in the regions 130–144 and 144–160, were most immunogenic; thus, it is highly possible that our predicted epitopes in these 2 regions are genuine CD8 T cell epitopes. Cell-mediated immunity is another arm of adaptive immunity that is of importance in host defense against PCV2 infection since PCV2 virus is an intracellular pathogen and has evolved to cause a persistent infection in immune cells [55]. A previous study demonstrated that the number of IFN-γ-secreting cells was inversely correlated with PCV2 viral loads in serum [56].

T cell epitope prediction of the PCV2 capsid proteins using two immunoinformatic tools, NetMHC and NetCTLpan, showed that the peptides predicted by NetCTLpan were more immunogenic compared to the peptides predicted by NetMHC. While the NetMHC tool has been underlined with the high, false positive rate of predicted epitopes [57], the NetCTLpan tool has been optimized to achieve high specificity [58]. Interestingly, the T cell epitopes predicted based on swine SLA could efficiently bind to mouse H-2 to stimulate mouse CD8 T cells. This implies a common characteristic of epitope recognition by MHC class I in swine (SLA) and mouse (H-2).

Our main goal in this study is to obtain a PCV2 vaccine candidate that provides broad immune responses against both PCV2a and PCV2b. As demonstrated by the results, our vaccines elicited antibody and T cell responses specific to both conserved and genotype-specific regions. Some immunodominant epitopes such as B cell epitope at residues 92–120 and T cell epitopes at residues 103–111 are identified as conserved sequences among other PCV2 genotypes such as the recently emerged PCV2d genotype (accession number AWD32058.1). Moreover, PCV2b-2a was designed from consensus sequences of Cap2a and Cap2b to cope with the large number of variants in PCV2a and PCV2b. With these attributes, the PCV2b-2a-based vaccines have the potential to provide broad protective immune response against not only PCV2a and PCV2b, but also other PCV2 genotypes. Moreover, our new bivalent PCV2 vaccine was developed based on a fusion protein of Cap2a and Cap2b, while other studies of bivalent PCV vaccines are based on a mixture of two monovalent vaccines. Thus, employing a fusion protein for bivalent vaccine development not only induces a broad immune response, but also reduces the cost of vaccine production as it requires only one round of vaccine preparation and validation.

## 5. Conclusions

In this study, we successfully developed a DNA-based bivalent PCV2 vaccine combining capsid protein from PCV2 genotypes 2a and 2b. The PCV2b-2a immunogen conjugating with fusion cytokines GM-CSF and IL-4 was the most immunogenic and induced pronounced PCV2-specific humoral and cellular immune responses in a mouse model. By coupling immunoinformatics with an immunoassay, we revealed the 3 most immunoreactive linear B cell epitopes and 5 putative CD8 T cell epitopes on Cap2a and Cap2b in the context of the PCV2b-2a immunogen. Despite a low neutralizing activity, sera from mice receiving the PCV2b-2a-based DNA vaccine could neutralize PCV2a with the endpoint titer of 8. Importantly, this bivalent vaccine induced broad responses that cover both PCV2a and PCV2b and may also cross-react with other PCV2 genotypes, as some immunogenic epitopes are well conserved among PCV2.

These results indicate that the PCV2b-2a immunogen holds promise as a new candidate for further PCV2 vaccine development, utilizing alternative platforms that offer greater efficiency than DNA vectors and are feasible for large-scale production at an affordable cost for pig producers. Moreover, the potency of the PCV2b-2a-based vaccine needs to be evaluated in pigs, the real target of this vaccine, before practical application can be suggested.

## Figures and Tables

**Table 1:** Primers used for PCR amplifications to generate bivalent gene fragments. Letters in bold are the restriction enzymes sites of *Pst*I and *Not*I.

Primer Name	Sequence (5′ to 3′)	Length (nt)
5′-flanking-RE-F	ATTGTCGACGGATCC**CTGCAG**GAAGCTTTTCCC	33
V5(PCV)FuF2A-F	AATCCTCTGCTGGGCCTCGACAGAGCCAAGAGAAGCGGCAGC	42
SS-PCV2b-F1	CCCCTGGTGAGCAGCCAGTGCGTGAACCTGATGACCTACCCTAGAAGGAGG	51
SS-PCV2b-F2	ATGTTCGTGTTCCTGGTGCTGCTGCCCCTGGTGAGCAGCCAGTGC	45
PstI-SS-F3	CCCTGCAGGAAGCTTTTCCCCGGGGCCACCATGTTCGTGTTCCTGGTGCTG	51
V5(PCV)FuF2A-R	GCTGCCGCTTCTCTTGGCTCTGTCGAGGCCCAGCAGAGGATT	42
Stop-RE-R	TAAGCCCGGG**GCGGCCGC**TTATCA	24

**Table 2:** Predicted B cell epitopes of the PCV2a and PCV2b capsid proteins.

No.	Name	Position	Genotype	Peptide	Length
1	Bc-Cap2a/2b-1	5–18	PCV2b/PCV2a	RRRYRRRRHRPRSH	14
2	Bc-Cap2a/2b-2	25–40	PCV2b/PCV2a	RRPWLVHPRHRYRWRR	16
3	Bc-Cap2b-3	59–70	PCV2b	RTTVKTPSWAVD	12
4	Bc-Cap2b-4	77–120	PCV2b	NDFLPPGGGSNPRSVPFEYYRIRKVKVEFWPCSPITQGDRGVGS	44
5	Bc-Cap2b-4.1	77–95	PCV2b	NDFLPPGGGSNPRSVPFEY	19
6	Bc-Cap2a/2b-4.2	92–120	PCV2b/PCV2a	PFEYYRIRKVKVEFWPCSPITQGDRGVGS	29
7	Bc-Cap2b-5	139–152	PCV2b	DPYVNYSSRHTITQ	14
8	Bc-Cap2b-6	162–182	PCV2b/PCV2a	TPKPVLDSTIDYFQPNNKRNQ	21
9	Bc-Cap2b-7	188–196	PCV2b	QTAGNVDHV	9
10	Bc-Cap2b-8	201–211	PCV2b	AFENSIYDQEY	11
11	Bc-Cap2a-3	57–67	PCV2a	VKATTVRTPSW	11
12	Bc-Cap2a-4	77–94	PCV2a	DDFVPPGGGTNKISIPFE	18
13	Bc-Cap2a-5	128–158	PCV2a	NFVPKATAQTYDPYVNYSSRHTIPQPFSYHS	31
14	Bc-Cap2a-6	167–182	PCV2b/PCV2a	LDSTIDYFQPNNKRNQ	16
15	Bc-Cap2a-7	188–196	PCV2a	QTSRNVDHV	9
16	Ref-1	26–36	PCV2b/PCV2a	RPWLVHPRHRY	11
17	Ref-2	117–131	PCV2b	GVGSSAVILDDNFVT	15
18	Ref-3	195–233	PCV2b	HVGLGTAFENSIYDQEYNIRVTMYVQFREFNLKDPPLNP	39

**Table 3:** Predicted CD8 T cell epitopes of the Cap2a and Cap2b proteins using NetCTLpan-1.1 and NetMHC-4.0. The prediction was performed with both swine SLA and mouse H-2 alleles.

Peptide Name	Start-End Position	Genotype	Peptide	Length	Allele-Binder	Prediction Tool
CapCTL-1	1–8	PCV2b, PCV2a	MTYPRRRY	8	SLA-1, SLA-3	NetCTLpan
CapCTL-2	89–96	PCV2b	RSVPFEYY	8	SLA-1, SLA-3	NetCTLpan
CapCTL-3	103–111	PCV2b, PCV2a	VEFWPCSPI	9	SLA-2	NetCTLpan
CapCTL-4	136–144	PCV2b	LTYDPYVNY	9	SLA-1, SLA-2	NetCTLpan
CapCTL-5	148–156	PCV2b	HTITQPFSY	9	SLA-1	NetCTLpan
CapCTL-6	148–156	PCV2a	HTIPQPFSY	9	SLA-1, SLA-2	NetCTLpan
CapCTL-7	150–160	PCV2b	ITQPFSYHSRY	11	SLA-1, SLA-2, SLA-3	NetCTLpan
CapCTL-8	48–55	PCV2b, PCV2a	RLSRTFGY	8	SLA-1, SLA-2	NetCTLpan
CapCTL-9	89–96	PCV2a	ISIPFEYY	8	SLA-1, SLA-3	NetCTLpan
CapCTL-10	130–138	PCV2b	VTKATALTY	9	SLA-1, SLA-2, SLA-3	NetCTLpan, NetMHC
CapCTL-11	136–144	PCV2a	QTYDPYVNY	9	SLA-1	NetCTLpan
CapCTL-12	144–154	PCV2b	YSSRHTITQPF	11	SLA-2	NetCTLpan
CapCTL-13	37–45	PCV2b/PCV2a	RWRRKNGIF	9	SLA-3	NetMHC
CapCTL-14	133–141	PCV2b	ATALTYDPY	9	SLA-1, SLA-2	NetMHC
CapCTL-15	166–174	PCV2b/PCV2a	VLDSTIDYF	9	SLA-1	NetMHC
CapCTL-16	85–94	PCV2a	GTNKISIPF	9	SLA-1	NetMHC
CapCTL-17	133–141	PCV2a	ATAQTYDPY	9	SLA-1, SLA-2	NetMHC
H2-Cap2b2a-1	81–93	PCV2b	PPGGGSNPRSVPF	13	H-2-Dd	NetMHC
H2-Cap2b2a-2	140–151	PCV2b/PCV2a	PYVNYSSRHTI	12	H-2-Kd	NetMHC
H2-Cap2b2a-3	159–171	PCV2b/PCV2a	RYFTPKPVLDSTI	13	H-2-Kd	NetMHC

## Data Availability

Correspondence and requests for additional materials and data should be addressed to Y.M.R.

## Supplementary Materials

The following supporting information can be downloaded at: https://www.mdpi.com/article/10.3390/vaccines12030324/s1. Table S1: Predicted physico-chemical and antigenicity properties of the consensus PCV2 capsid protein and designed immunogen in comparision to natural PCV2 capsid proteins; Figure S1: Generated consensus amino acid sequences of the PCV2a and PCV2b capsid (Cap2a and Cap2b) proteins; Figure S2: Amino acid sequences of the bivalent immunogen Cap2b-2a. Linker is indicated in blue; V5 tag at the C-terminus is indicated in red; Figure S3: Expression of capsid proteins of PCV2a and PCV2b in E. coli. E. coli strain BL21(DE3) pLberaysS bearing recombinant pET28b plasmid was grown and induced with 1 mM IPTG for 3 h at 37 °C; Figure S4: Optimization of protein amount used in ELISA. ELISA was performed with a 1:100 diluted serum from mice receiving vaccine and PBS; Figure S5: 3-D structure of the PCV2 cap proteins labeled with the immunodominant linear B-cell epitopes; Figure S6: Correlation of the T cell response and antibody response (total IgG) of all four groups of the immunized mice.
