# Supplementary material for: Humoral and Cellular Immune Responses Induced by Bivalent DNA Vaccines Expressing Fusion Capsid Proteins of Porcine Circovirus Genotypes 2a and 2b
Source: Vaccines (Basel). 2024 Mar 18;12(3):324. doi: 10.3390/vaccines12030324 (PMC10974225; doi:10.3390/vaccines12030324)
Supplement: Supplementary file 1 [file vaccines-12-00324-s001.zip › vaccines-2833822-supplementary.pdf]

Table S1. Predicted physico-chemical and antigenicity properties of the consensus PCV2 capsid protein and designed immunogen in comparision to natural PCV2 capsid proteins.

Property	PCV2a Cap (Acc. No. AIP98369.1)	PCV2b Cap (Acc. No. ACQ45276.1)	Consensus Cap2a	Consensus Cap2b	Bivalent immunogen PCV2b-2a
**1. Number of amino acid residues**	233	233	233	233	486
**2. Molecular weight (kDa)**	27.9	27.9	27.9	27.9	57.5
**3. Isoelectric point (PI)**	10.68	10.83	10.69	10.75	10.68
**4. Estimated half-life**	> 30 hours in mammalian reticulocytes, in vitro > 20 hours in yeast, in vivo). > 10 hours in *E.coli*, in vivo	> 30 hours in mammalian reticulocytes, in vitro > 20 hours in yeast, in vivo). > 10 hours in *E.coli*, in vivo	> 30 hours in mammalian reticulocytes, in vitro > 20 hours in yeast, in vivo). > 10 hours in *E.coli*, in vivo	> 30 hours in mammalian reticulocytes, in vitro > 20 hours in yeast, in vivo). > 10 hours in *E.coli*, in vivo	> 30 hours in mammalian reticulocytes, in vitro > 20 hours in yeast, in vivo). > 10 hours in *E.coli*, in vivo
**5. Aliphatic index**	62.23	63.09	62.66	63.09	64.12
**6. Instability index**	60.53	59.81	57.84	59.69	57.35
**7. GRAVY**	-0.828	-0.833	-0.875	-0.830	-0.816
**8. Antigenicity by Vaxigen v2.0 (threshold > 0.4)**	0.557	0.615	0.601	0.619	0.604

Consensus Cap2a

MTYPRRRYRRRRHRPRSHLGQILRRRPWLVHPRHRYRWRRKNGIFNTRLSRTFGYTV-
KATTVRTPSWAVDMLRFNLDDFVPPGGGTNKISIPFEYYRIRKVKVEFWPCSPITQGDRGVGSTAVILDDNFVPKATAQTYDPYVN
YSSRHTIPQPFSYHSRYFTPKPVLDSTIDYFQPNNKRNQLWLRLQTSRNVDHVGLGTAFEN-
SKYDQDYNIRVTMYVQFREFNLKDPPLKP

Consensus Cap2b

MTYPRRRYRRRRHRPRSHLGQILRRRPWLVHPRHRYRWRRKNGIFNTRLSRTFGYTIKRT-
TVKTPSWAVDMMRFNINDFLPPGGGSNPRSVPFEYYRIRKVKVEFWPCSPITQGDRGVGSSAVILDDNFVTKATALTYDPYVNYSS
RHTITQPFSYHSRYFTPKPVLDSTIDYFQPNNKRNQLWLRLQTAGNVDHVGLGTAFEN-
SIYDQEYNIRVTMYVQFREFNLKDPPLNP

Figure S1. Generated consensus amino acid sequences of the PCV2a and PCV2b capsid (Cap2a and Cap2b) proteins.

MTYPRRRYRRRRHRPRSHLGQILRRRPWLVHPRHRYRWRRKNGIFNTRLSRTFGYTIKRTTVKTPSWAVDM
MRFNINDFLPPGGGSNPRSVPFEYYRIRKVKVEFWPCSPITQGDRGVGSSAVILDDNFVTKATALTYDPYVN
YSSRHTITQPFSYHSRYFTPKPVLDSTIDYFQPNNKRNQLWLRLQTAGNVDHVGLGTAFENSIYDQEYNIRV
TMYVQFREFNLKDPPLNP
GGGEAAAKGGG
MTYPRRRYRRRRHRPRSHLGQILRRRPWLVHPRHRYRWRRKNGIFNTRLSRTFGYTVKATTVRTPSWAVD
MLRFNLDDFVPPGGGTNKISIPFEYYRIRKVKVEFWPCSPITQGDRGVGSTAVILDDNFVPKATAQTYDPYV
NYSSRHTIPQPFSYHSRYFTPKPVLDSTIDYFQPNNKRNQLWLRLQTSRNVDHVGLGTAFENSKYDQDYNIR
VTMYVQFREFNLKDPPLKP
IPNPLLGLD

Figure S2. Amino acid sequences of the bivalent immunogen Cap2b-2a. Linker is indicated in blue; V5 tag at the C-terminus is indicated in red.

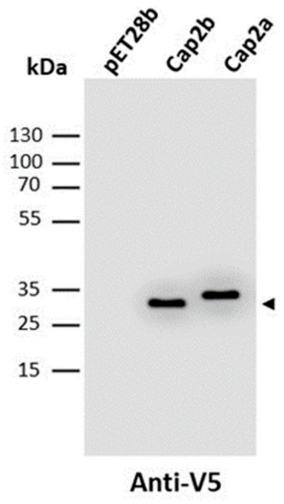

Figure S3. Expression of capsid proteins of PCV2a and PCV2b in *E. coli*. *E. coli* strain BL21(DE3) *pLberaysS* bearing recombinant pET28b plasmid was grown and induced with 1 mM IPTG for 3 hours at 37 °C. The cells were harvested and lysed. Only soluble fraction was used to confirm the protein expression by Western blot using anti-V5 antibody as a primary antibody. Different gene constructs used in the transformation are indicated on the top of each well. Predicted sizes of Cap2a and Cap2b are depicted.

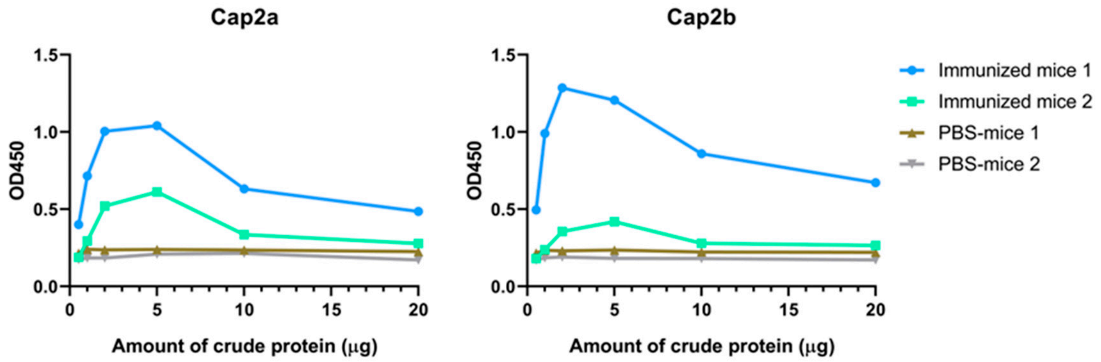

Figure S4. Optimization of protein amount used in ELISA. ELISA was performed with a 1:100 diluted serum from mice receiving vaccine and PBS. Various amounts of the Cap2a and Cap2b proteins in *E. coli* lysate (0.5 μg, 1 μg, 2 μg, 5 μg, 10 μg, and 20 μg/well) were tested.

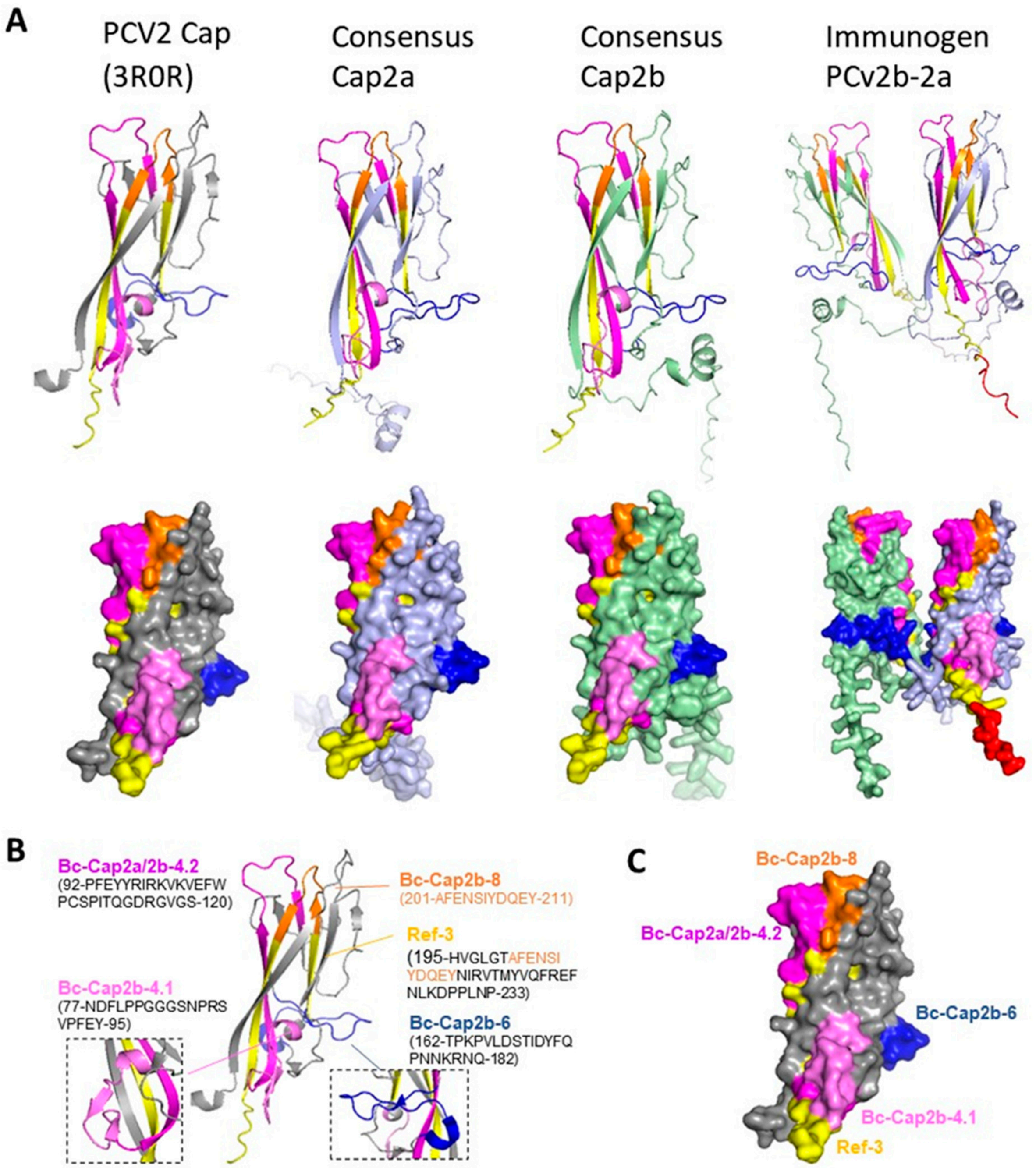

Figure S5. 3-D structure of the PCV2 cap proteins labeled with the immunodominant linear B-cell epitopes. **(A)** Predicted 3-D structures of the monomeric consensus Cap2a and Cap2b proteins in comparison to 3-D structure of the PCV2 capsid protein from database (PDB 3R0R). 3-D structures of the consensus Cap2a and Cap2b proteins were predicted using the ColabFold v1.5.5: AlphaFold2 online server. Top panel presents 3-D structure with secondary structure composition; bottom panel presents surface of the proteins. **(B)** Location and secondary composition of the immunodominant linear B-cell epitopes on the PCV2 capsid protein (PDB 3R0R). Each epitope is labeled with different colors. **(C)** Surface exposure of each immunodominant linear B-cell epitope on the PCV2 Cap protein.

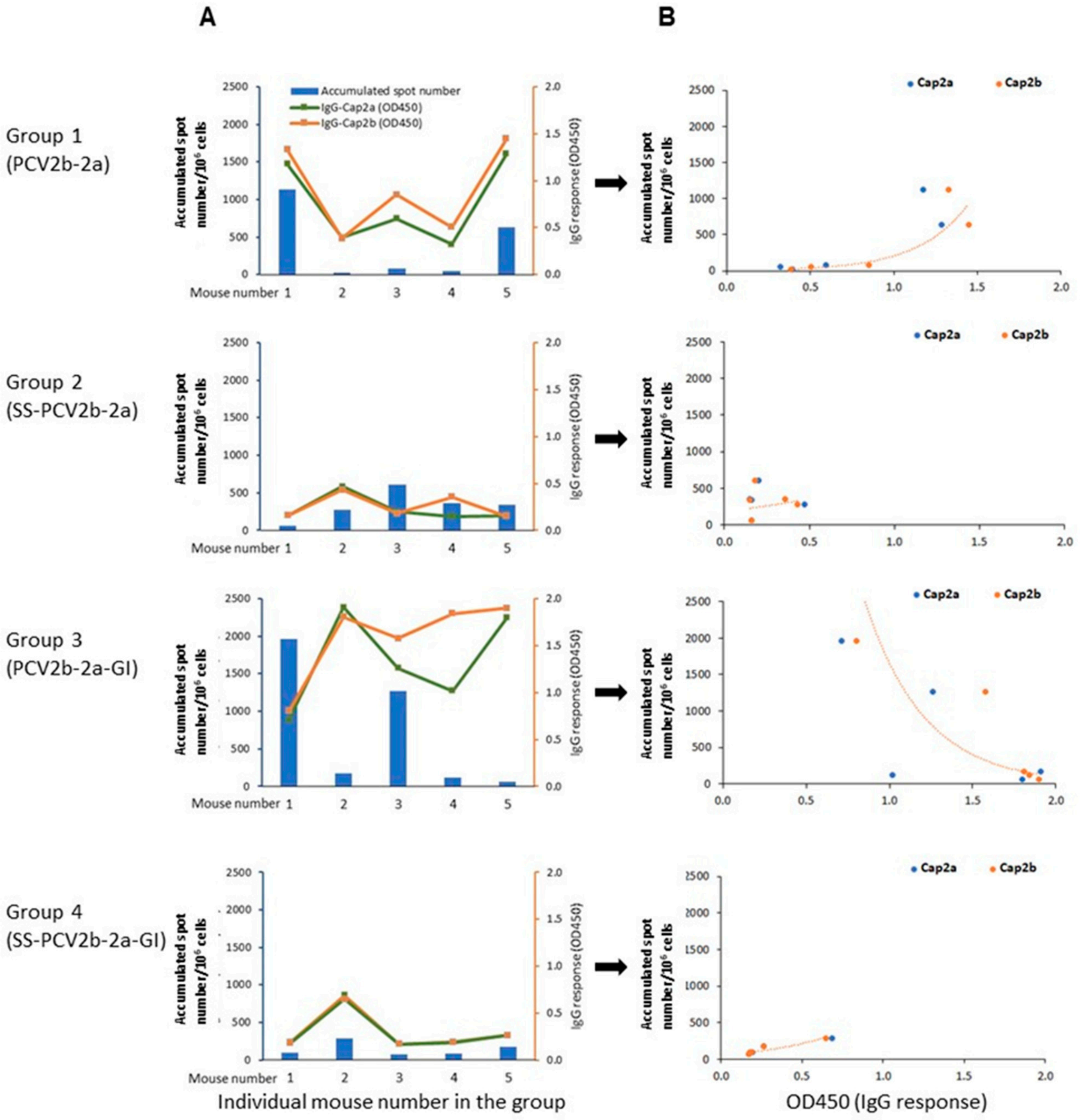

Figure S6. Correlation of the T cell response and antibody response (total IgG) of all four groups of the immunized mice. **(A)** Graphs combining T cell response (accumulated spot number in primary Y-axis) and total IgG response (OD450 in secondary Y-axis) of each mouse in the group. **(B)** correlation of the T cell response (X-axis) and antibody response (Y-axis). Orange line is a trend line indicating correlation between the T cell response and antibody response.
